# Identification of Serum-Based Metabolic Feature and Characteristic Metabolites in Paraquat Intoxicated Mouse Models

**DOI:** 10.3389/fphys.2020.00065

**Published:** 2020-02-06

**Authors:** Youjia Yu, Zishan Gao, Jiaqian Lou, Zhengsheng Mao, Kai Li, Chunyan Chu, Li Hu, Zheng Li, Chuwei Deng, Hanting Fan, Peng Chen, Huijie Huang, Yanfang Yu, Jingjing Ding, Ding Li, Feng Chen

**Affiliations:** ^1^Department of Forensic Medicine, School of Basic Medical Science, Nanjing Medical University, Nanjing, China; ^2^Clinical Acupuncture and Moxibustion Department, Second School of Clinical Medicine, Nanjing University of Chinese Medicine, Nanjing, China; ^3^Key Laboratory of Targeted Intervention of Cardiovascular Disease, Collaborative Innovation Center for Cardiovascular Disease Translational Medicine, Nanjing Medical University, Nanjing, China

**Keywords:** paraquat, intoxication, metabolomics, 3-indolepropionic acid, 2-hydroxybutyric acid, the ratio of L-serine/glycine

## Abstract

Paraquat (PQ) is a widely used herbicide which can cause high mortality to humans. However, relatively few studies focus on metabolic feature of PQ intoxication for investigating the underlying mechanisms. Here we performed non-targeted metabolomics profiling of serum samples from acute and chronic PQ intoxicated mouse models by gas chromatography time-of-flight mass spectrometry (GC–TOF/MS) to identify metabolic feature and characteristic metabolites of acute and chronic PQ intoxication. Results showed that 3-indolepropionic acid (IPA) and pathway of glycine, serine, and threonine metabolism were significantly altered after acute PQ intoxication; 2-hydroxybutyric acid and the ratio of L-serine/glycine were of significance between acute and chronic PQ intoxication. Then targeted metabolomics profiling was conducted by liquid chromatography–mass spectrometry (LC–MS) analysis to confirm the changes of IPA after acute PQ intoxication. Moreover, IPA-producing gut bacteria in feces were quantified by qRT-PCR to explain the varied IPA serum concentration. *Clostridium botulinum* and *Peptostreptococcus anaerobius* were significantly suppressed after acute PQ intoxication. The data suggested that PQ caused oxidative damage partially through suppression of anti-oxidative metabolite producing gut bacteria. In conclusion, we identified characteristic metabolites and pathway of acute and chronic PQ intoxication which could be potential biomarkers and therapeutic targets.

## Introduction

Paraquat (PQ, *N*,*N*′-dimethyl-4,4′-bipyridinium dichloride) is the most widely used herbicide for its ability in rapid contact-dependent killing of broad leaf weeds and plants (Suntres, 2002). Although the usage of PQ has been banned in many countries worldwide and recently in China due to its potential danger to human health, PQ intoxication, either by suicidal ingestion or occupational exposure, was reported to be frequently occurred and resulted in a fatality 1rate of 38.08% in China (Yin et al., 2013). Acute intoxication caused by PQ ingestion leads to multiple organ damage especially in the lungs, which is characterized by severe pulmonary inflammation, edema, and epithelial cell destruction. Progressive pulmonary fibrosis is the most serious consequence caused by PQ, and is often associated with high mortality due to the lack of effective therapeutic strategy (Suntres, 2002; Hu et al., 2016). However, the underlying mechanism of PQ intoxication remains elusive, resulting in a limitation of proper treatment. Moreover, PQ intoxication is easy to be overlooked by physicians due to the similarities of symptoms with other diseases, which leads to delayed diagnosis and therapy. Thus, it is of great importance and urgency to identify diagnostic biomarkers and develop effective therapeutic approaches for PQ intoxication.

In recent years, the rapid development of chemical analysis techniques benefits the applications of multi-omics-based approaches in medical research. Metabolomics had been recognized as an important and powerful tool to study human diseases. Gas chromatography-time-of-flight mass spectrometry (GC-TOF/MS) is one of the most widely used platforms in non-targeted metabolomics studies (Lenz and Wilson, 2007), which provides high chromatographic resolution, high mass measurement accuracy, and rapid spectrum acquisition (Ni et al., 2016). Given the poor prognosis of PQ intoxication, previous studies focused on mining potential prognosis biomarkers from patient samples by either metabolomics or other non-high-throughput approaches (Yeo et al., 2012; Wunnapuk et al., 2013; Wan et al., 2018; Chen et al., 2019a). Relatively few studies paid attention to the metabolic feature caused by PQ exposure to help us investigate the underlying mechanisms, which might contribute to develop new therapeutic targets and diagnosis biomarkers.

In the present study, we used a GC-TOF/MS-based technique (Ni et al., 2016) following machine learning statistic methods to identify metabolic features in our well-established acute and chronic PQ intoxicated mouse models (Hu et al., 2016), aiming at revealing metabolic feature and the alterations of metabolic pathways, thus providing new points of view for understanding the pathogenesis and toxicological characteristics of PQ-induced organ damage. Then we performed targeted metabolomics to determine serum concentrations of characteristic metabolite and verify the results of non-target metabolomics by using liquid chromatography–mass spectrometry (LC–MS) system. Furthermore, we quantified gut bacteria in feces samples that might be responsible for the alterations of serum metabolite. Our study helped to reveal new mechanism of PQ intoxication through identification of metabolic feature and characteristic metabolites.

## Materials and Methods

### Mouse Models

This study was designed in accordance with the guidelines of Institute for Laboratory Animal Research of the Nanjing Medical University. All protocols were approved by the Animal Care and Ethical Committee of Nanjing Medical University. Thirty 7–8 weeks male mice weighing 20–25 g (C57/BL6 mice, Oriental Bio Service Inc., Nanjing, China) were randomly divided into three groups. Mice were maintained under a constant environmental condition with temperature 23 ± 2°C, humidity 55 ± 5%, and 12:12 h light/dark cycle in the Animal Research Center of Nanjing Medical University with free access to food and water before and after all procedures.

For acute intoxication model, 10 mice were treated with single intraperitoneal (i.p.) injections of PQ (856177, Sigma, St. Louis, MO, United States) 50 mg/kg on day 0. Among them, eight mice survived at the time of sample collection and were sacrificed on day 3. For chronic PQ intoxication model, 10 mice were treated with single i.p. injections of PQ 10 mg/kg on day 0 and were maintained until sacrificed on day 30 (Hu et al., 2016). Ten control mice were injected with the same volume of vehicle. All mice in the chronic and control groups were alive before sacrifice.

### Sample Collection and Preparation

Peripheral blood was obtained from mice at the end of study and was incubated for 30 min at room temperature to allow for clotting then centrifuged at 2,500 × *g* for 10 min to extract the serum from clot. Serum aliquots were stored at −80°C until the time of analysis. Feces samples were collected from ileocecal junction after sacrifice and stored at −80°C until DNA extraction from feces.

The serum sample preparation procedures were described previously (Wang et al., 2013). In brief, the serum samples were centrifuged at 4°C, 3,000 × *g* to separate debris or lipid layer. Each 50 μl aliquot was mixed with 10 μl internal standard, and 175 μl pre-chilled methanol was added. Then the mixtures were kept at −20°C for 20 min and centrifuged for 20 min at 4°C 14,000 × *g*. The supernatant was transferred into an autosampler vial (Agilent Technologies, Foster City, CA, United States) separately and was evaporated briefly, following by lyophilizing with a FreeZone freeze dryer (Labconco, Kansas City, MO, United States). The dried samples were then derivatized with 50 μl methoxyamine at 30°C for 2 h, followed by adding 50 μl *N*-methyl-*N*-trimethylsilyl-trifluoroacetamide (MSTFA) containing fatty acid methyl esters (FAMEs) as retention indices and further incubating at 37.5°C for 1 h. Finally, the derivatized samples were injected into the GC-TOF/MS for analysis.

### Metabolite Measurements by Non-targeted Metabolomics Profiling

The non-targeted metabolomics profiling was performed on XploreMET^TM^ platform (Metabo-Profile, Shanghai, China) with a GC-TOF/MS system (Pegasus HT, Leco Corp., St. Joseph, MO, United States) with an Agilent 7890B gas chromatography and a Gerstel multipurpose sampler MPS2 with dual heads (Gerstel, Mülheim, Germany). A Rxi-5ms capillary column (30 m × 250 μm i.d., 0.25-μm film thickness; Restek Corporation, Bellefonte, PA, United States) was used for separation. Helium was used as the carrier gas at a constant flow rate of 1.0 ml/min. The temperature of injection was set to 270°C. The source temperature was 220°C. The measurements were made using electron impact ionization (70 eV) in the full scan mode (*m*/*z* 50–500).

### Statistical Data Analysis

The raw data generated by GC-TOF/MS were processed using ADAP-GC 3.0 for automated baseline denoting and smoothing, peak picking and deconvolution, creating reference database from the pooled QC samples, metabolite signal alignment, missing value correction and imputation, and QC correction (Ni et al., 2016). The Human Metabolome Database^1^ (Wishart et al., 2018) was used as an additional reference source. Moreover, ratio of the two adjacent metabolites from KEGG database^2^ was calculated.

Before statistical analysis, the resulting integral data were log-transformed and Pareto-scaled, for the purpose of adjusting skewed distributions in metabolomics data. To differentiate the change of metabolic profiling among three groups, principal component analysis (PCA) and partial least square discriminate analysis (PLS-DA) were firstly conducted using MetaboAnalyst 4.0^3^ (Chong et al., 2018). Data were showed by the principal component (PC) score and loading plots. Each point on the scores plot indicated an individual sample, and each point on the loadings plot indicated a single peak area. Orthogonal signal correction (OSC) was further executed to minimize the variations not correlated to group membership and to maximize the separation, followed by PLS analysis.

To investigate potential biomarkers for PQ intoxication, we performed Lasso penalized regression using glmnet package in R. The overall penalty parameter α was set to 1 in terms of selecting those with highest predict value of metabolites in glmnet. Tenfold internal cross-validation was executed to validate the regression model and to achieve the minimum λ, and subsequently selected most relevant metabolites for PQ intoxication using this minimum λ. After the generation of final list of potential metabolite biomarkers by Lasso, pair-wised *t*-test was conducted on those metabolites to gain the correct *P-*value by embayed t.test function in R. Bonferroni correction was performed on the result *P-*values by using p.adjust function in R, thus the adjusted *P-*value of selected metabolites was finally acquired.

To elucidate potential significant metabolic pathways for PQ intoxication, metabolic pathway enrichment analysis (MPEA) was performed using MetaboAnalyst 4.0. False discovery rate (FDR) correction was conducted automatically at MetaboAnalyst 4.0 to validate the significant metabolic pathway.

Diagnostic abilities of the potential biomarkers were assessed by receiver operating characteristic (ROC) curve analysis according to the following criteria: the area under the curve (AUC) of 0.9–1.0 indicated excellent performance, 0.8–0.89 good performance, 0.7–0.79 fair performance, 0.6–0.69 poor performance, and <0.6 insignificant value (Haase-Fielitz et al., 2009). These statistical analyses were conducted by using GraphPad Prism version 6.01 (GraphPad Software, United States) as described previously (Yu et al., 2016; He et al., 2017).

### Quantification of Serum 3-Indolepropionic Acid Concentrations by Targeted Metabolite Profiling

The standard stock solution of 3-indolepropionic acid (IPA) (V900491, Sigma, United States) at 1 mg/ml was prepared in methanol. Calibration standards were made at 1.00, 2.50, 5.00, 10.0, 25.0, and 50.0 ng/ml for IPA. An aliquot of 50 μl of serum sample was pipetted into a 1.5-ml eppendorf tube. Then 100 μl of methanol was added and vortex mixed, subsequently centrifuged at 12,000 × *g* for 5 min. Finally, an aliquot of 2 μl of the clear upper layer was injected into the LC–MS system. Chromatographic separation was performed on an Agilent Poroshell 120 SB-Aq (100 mm × 2.1 mm, 2.7 μm) column under ambient conditions. The mobile phase was composed of 0.05% formic acid in water (A) and 0.05% formic acid in acetonitrile (B) and kept at a flow rate of 0.3 ml/min. Gradient conditions started with 10% B for 1 min, increased to 90% B in 16 min, and then held for 4 min, returned to initial conditions in 0.1 min and re-equilibrated for 2 min. The LC–MS analysis was performed on a TripleTOF^®^ 5600+ mass spectrometer with electrospray ionization (ESI) in the positive ionization mode with selective ion monitoring (SIM) at *m*/*z* 190.0853 for IPA. Source parameters were: curtain gas, 48 psi; temperature, 630°C; ion spray voltage, 4.2 kV; declustering potential, 70 V.

### Gut Bacteria Genomes Isolation and Quantification of IPA-Producing Gut Bacteria

Gut bacteria genomes were isolated from equal weight of feces samples of control and acute groups with TIANamp Stool DNA Kit (Tiangen, Beijing, China) as manufactory’s instruction. Six IPA-producing bacteria from literatures were quantified by qRT-PCR by using Fast super EvaGreen qPCR Master Mix (US Everbright Inc., United States) according to manufactory’s instruction. Primers were as follows (5′–3′): for *Clostridium sporogenes*: F: AAGAACACCAGTGGCGAAGG, R: GTTTACGGCGTGG ACTACCA; for *Clostridium botulinum*: F: CACATGCA AGTCGAGCGATG, R: AGGCTTTCCCCCACTTTGAG; for *Clostridium cadaveris*: F: AAATACCCGGGCTCAACCTG, R: CCTCAGTGTCAGTTACAGTCCA; for *Peptostreptococcus anaerobius*: F: TTTATGAGAGTTTGATCCTGGCT, R: GTGTA TAGGGCAGGTTACCCA; for *Peptostreptococcus russellii*: F: AA CCGCAAGGAAGAAGTCGT, R: CACCTTCCGATACGGCTA CC; and for *Peptostreptococcus stomatis*: F: GACTGAGGTGA CAGGTGGTG, R: CCCAACTGAATGCTGGCAAC. PCR products for different bacteria were cloned into vector to generate standard curves separately. Copies of each bacterium per gram of feces in each sample were calculated according to standard curves. Statistical analyses were performed by using GraphPad Prism version 6.01.

## Results

### Metabolomics Profiling of Serum Samples From Acute, Chronic PQ Intoxicated, and Control Mice

**FIGURE 1:**
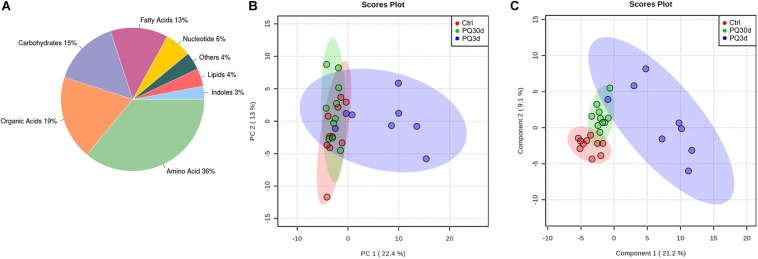
Overviews of metabolic profiles. **(A)** Metabolite classes and compositions detected in the samples. Overview of metabolic profiles of Ctrl, PQ30d, and PQ3d groups using principal component analysis (PCA) **(B)** and partial least squares discriminant analysis (PLS-DA) **(C)** score plots for serum samples analyzed by GC-TOF/MS.

A total of 28 serum samples from acute group (PQ3d), chronic group (PQ30d), and control group (Ctrl) were analyzed using non-targeted metabolomics profiling and 150 metabolites were detected, among which 107 were annotated by ADAP-GC 3.0 using a strict matching algorithm (Supplementary Table 1_v1). Twenty-five ratios of metabolites from KEGG metabolic pathways were measured. However, 43 metabolites remained currently unidentified. The annotated metabolites and their chemical classes were illustrated in Figure 1A.

The overview of global metabolic profiles according to the quantitative results for the metabolites in the mice serum samples, as revealed by PCA scores plots (Figure 1B), showed obvious dissimilarities between the PQ3d group and the Ctrl group or the PQ30d group. This differentiation could be described by the first PC 1, which accounted for 22.4% of the variance. The second PC (PC 2) accounted for 13.0% of the variance. PLS-DA could be utilized to facilitate the screening of metabolic biomarkers. The PLS-DA scores plot for the three data sets demonstrated good separation trend among Ctrl, PQ3d, and PQ30d groups (Figure 1C). The components 1 and 2 described 21.2 and 9.1% of the variance, separately.

### Metabolomics Alteration in Serum Induced by PQ Intoxication

**FIGURE 2:**
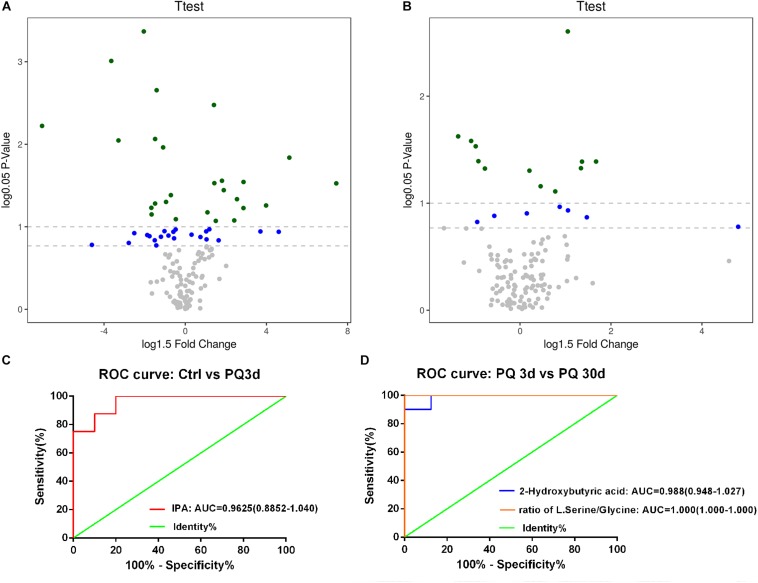
Identification and performance assessment of metabolites with significance. An enhanced volcano plot showed the differential metabolites selected with multi-criteria assessment from PQ3d vs. Ctrl **(A)** and PQ30d vs. Ctrl **(B)**. The *P*-value together with log 1.5 fold change (FC) was introduced with a cutoff value of 0.05, 0.1 for *P*-value and 1.5 for log 1.5 FC, respectively. ROC curve of IPA distinguishing PQ3d vs. Ctrl **(C)**, 2-hydroxybutyric acid, and the ratio of L-serine/glycine **(D)** distinguishing PQ3d vs. PQ30d. The AUC values of each metabolite were shown below each ROC curve. Confidence interval (CI) was enclosed in parentheses after AUC value.

Based on the serum samples from mouse models of acute and chronic PQ intoxication, we identified 19 metabolites and 4 metabolite ratios differed significantly between the PQ3d group and the Ctrl group; 3 metabolites and 3 metabolite ratios differed significantly between the PQ30d group and the Ctrl group; and 20 metabolites and 7 metabolite ratios differed significantly between the PQ3d group and the PQ30d group. Significantly differentiated metabolites were listed in Supplementary Tables S1–S3. The enhanced volcano plot showed the differential metabolites selected with multi-criteria assessment (Figures 2A,B). The *P*-value together with log fold change (FC) was introduced with a cutoff value of 0.05, 0.1 for *P-*value and 1.5 for log FC, respectively.

For further identification of characteristic metabolites for acute and chronic PQ intoxication, we pairwise compared the data of each group by Bonferroni correction following Student’s *t*-test. Lasso regression was applied for feature selection to identify metabolites with significant FC. Results were listed in Supplementary Tables S4–S6. From comparison between PQ3d and Ctrl group, we identified IPA as a potential biomarker of acute PQ intoxication (*P* < 0.05 by both Bonferroni correction and coefficient = −0.77 by Lasso regression). IPA and the ratio of L-serine/glycine were identified as metabolites with significance in PQ3d vs. PQ30d (*P* < 0.05 by Bonferroni correction). The ratio of L-serine/glycine with coefficient = 1.43, but not IPA, was identified by Lasso regression as a potential biomarker for differentiating acute and chronic PQ intoxication. Moreover, we noticed 2-hydroxybutyric acid with coefficient = 1.70 by Lasso regression which was not validated by Bonferroni correction. This result of no significance might be due to the multicollinearity between metabolites and the inter-metabolite relationships within the metabolic network. Thus, we further performed correlation analysis on PQ3d vs. PQ30d and observed positive correlation between 2-hydroxybutyric acid and the ratio of L-serine/glycine (*R* = 0.73) (Supplementary Figure S1). The correlation of two metabolites might impact the result of Bonferroni correction. Since both 2-hydroxybutyric acid and L-serine/glycine showed statistically significant difference between PQ3d vs. PQ30d after FDR correction, these results indicated that these two metabolites could be potential biomarkers for distinguishing acute and chronic PQ intoxication. No metabolite with significance was identified from PQ30d vs. Ctrl after Bonferroni correction.

The performances of IPA, 2-hydroxybutyric acid, and the ratio of L-serine/glycine were assessed by ROC curve. The diagnostic ability was illustrated by AUC (Figures 2C,D). AUC values of all three metabolites or ratio were >0.9, indicating excellent performances. The results demonstrated that IPA could be taken as a potential biomarker for diagnosing acute PQ intoxication, and 2-hydroxybutyric acid and the ratio of L-serine/glycine could be potential biomarkers for differentiating acute and chronic PQ intoxication.

### Enrichment Analysis of Metabolic Pathway

**FIGURE 3:**
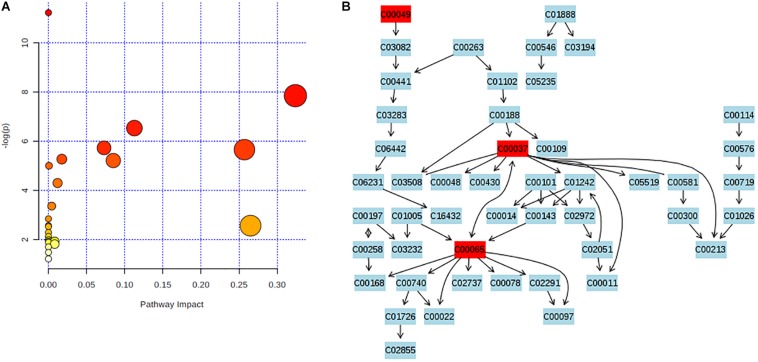
Enrichment analysis of metabolic pathway. **(A)** *P*-values were obtained by Holm–Bonferroni correction and FDR correction. The rightmost dot was the pathway of glycine, serine, and threonine metabolism. **(B)** Glycine, serine, and threonine metabolism was identified as metabolite pathway with significance in PQ3d vs. Ctrl. Identified metabolites involved in this pathway were highlighted in red. C00049: L-aspartic acid; C00037: glycine; C00065: L-serine.

Significantly altered metabolic pathways in acute or chronic PQ intoxication group comparing to control group were analyzed by using Metaboanalysist 4.0. The original and adjusted *P-*values together with impact factors were listed in Supplementary Tables S7, S8. We identified glycine, serine, and threonine metabolism as significant metabolite pathway in PQ3d vs. Ctrl with impact factors of 0.32 and *P* < 0.05 after both Holm–Bonferroni correction and FDR correction (Figures 3A,B). However, no significant metabolic pathway was identified in PQ30d vs. Ctrl.

### Determination of IPA Concentrations in Mice Serum by Targeted Metabolite Profiling

**FIGURE 4:**
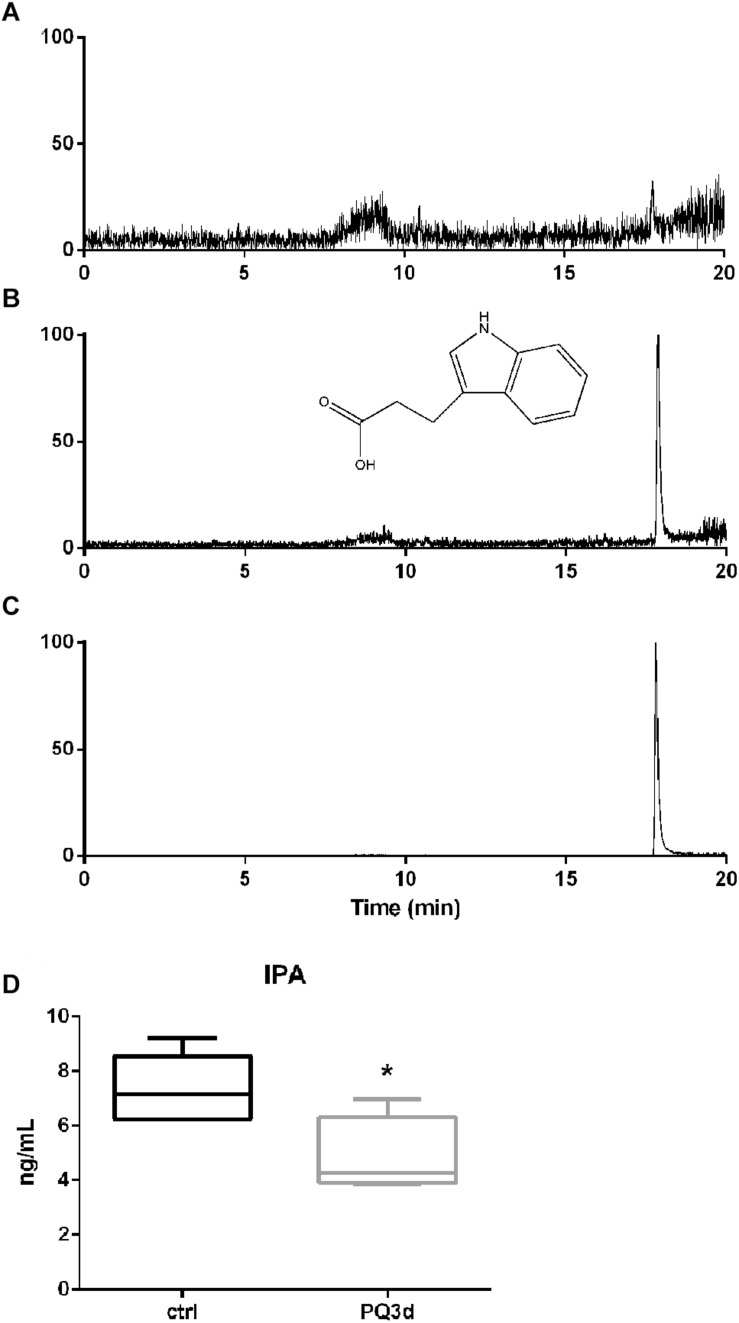
IPA concentrations in mice serum were determined by targeted metabolite profiling. **(A)** Typical SIM chromatograms of IPA in blank sample. **(B)** Blank sample spiked with IPA at LLOQ at 1.00 ng/ml. **(C)** Real serum sample obtained from a mice of the acute group. **(D)** IPA serum concentrations in Ctrl group and acute PQ intoxication group. *P < 0.05.

The calibration curves were linear over the concentration range of 1.00–50.0 ng/ml for IPA (*r*^2^ > 0.9998) with the lower limit of quantification (LLOQ) at 1 ng/ml (Figures 4A,B). The intra and inter-day precision and accuracy of analysis of the quality control samples were ≤12.7%. The matrix effects (mean ± RSD) were 96.4 ± 8.2%, indicating that no significant matrix effect was observed for the analyte. The method was successfully applied to quantify serum IPA concentrations of the acute and control groups (Figure 4C). IPA serum concentration was 7.37 ± 1.25 ng/ml in Ctrl group and significantly decreased to 4.84 ± 1.43 ng/ml in acute group (*P* < 0.05) (Figure 4D).

### Quantification of IPA-Producing Gut Bacteria

**FIGURE 5:**
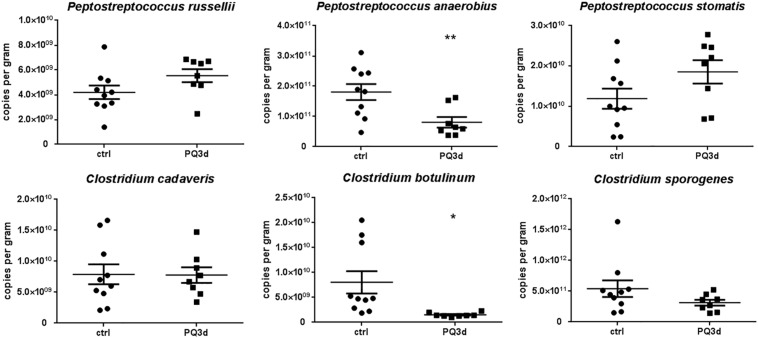
qRT-PCR results showed the feces content alterations of six IPA-producing gut commensal bacteria reported by literatures in control and acute PQ intoxication groups. *P < 0.05, **P < 0.01.

For clarifying bacteria that might be responsible for decreased IPA concentrations in serum after acute PQ intoxication, we performed qRT-PCR to quantify IPA-producing gut bacteria that reported by literatures. Among all six bacteria, *C. botulinum* and *P. anaerobius* were identified to be significantly suppressed during acute PQ intoxication, while levels of *P. russellii*, *P. stomatis*, *Clostridium cadaveria*, and *C. sporogenes* stayed unchanged (Figure 5).

## Discussion

**FIGURE 6:**
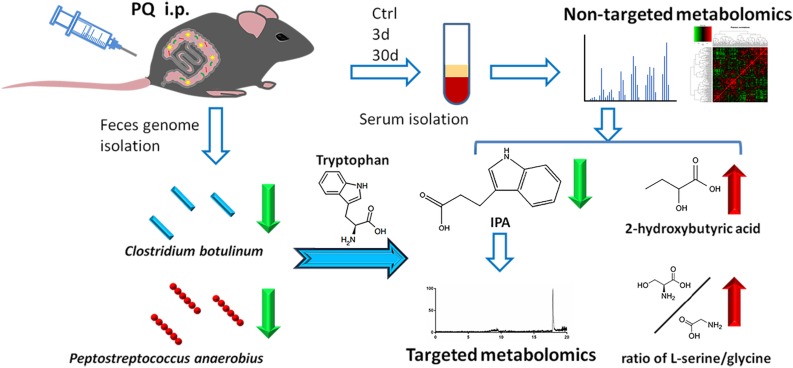
Work flow of the presented study.

In this study, we presented the first metabolomics profiling of both acute and chronic PQ intoxicated mouse models to our knowledge. We tried to mimic clinical features of PQ intoxication by injecting different doses of PQ to establish acute and chronic models. In clinical practice, patients who expose to high dose of PQ will pass away within a few days due to multi-organ injury or pneumonia; other patients who expose to low dose of PQ usually have no symptom at the beginning but suffer from pulmonary fibrosis later and pass away after about 1 month (Chen et al., 2019b). By using GC-TOF/MS technique following machine learning statistic methods, we observed significant metabolic features in mice serum after acute PQ intoxication while the metabolic alteration after chronic PQ intoxication was not obvious. We applied Bonferroni correction to adjust the *P-*values. Bonferroni correction is a popular method which compensates for multiple comparisons by dividing the significance level by the number of comparisons (Henry, 2015) and shows advantage of decreasing the risk of type I error (Armstrong, 2014). After that identification of characteristic metabolites was conducted by Lasso regression, which was targeted more on prediction due to its ability to exclude metabolites from the statistical model with a low association with assessed outcome (Pripp and Stanisic, 2017). IPA, 2-hydroxybutyric acid, and the ratio of L-serine/glycine were identified as significant metabolites or metabolite ratio. Results showed IPA with a negative coefficient by Lasso regression, indicating that decreased IPA concentration could prompt higher probability of acute PQ intoxication. Decreased serum concentrations of IPA in acute PQ intoxication groups were determined by a LC–MS-based target metabolomics profiling and decreased levels of IPA-producing bacteria *C. botulinum* and *P. anaerobius* were identified in feces (Figure 6).

Paraquat induces oxidative damage and cell death by generation of reactive oxygen species (ROS) including hydrogen peroxide, superoxide anion, and hydroxyl radicals (Fan et al., 2018; Chen et al., 2019a), and induction of cellular apoptosis (Kong et al., 2019). PQ toxicity is related to its capacity to capture electrons in cellular redox reactions and inhibit the reduction of NADP^+^ to NADPH in the cytosolic fraction, which induce oxidative stress by the overproduction of superoxide anion (O^2–^) (Baltazar et al., 2014; Blanco-Ayala et al., 2014). Interestingly, IPA, also known as indole-3-propionic acid, is a type of tryptophan catabolite to eliminate hydroxyl radical and alleviate oxidative stress-induced damage to variety of tissues (Karbownik et al., 2005; Wikoff et al., 2009; Zhang and Davies, 2016), which means IPA could be an endogenous anti-oxidative metabolite. IPA is only synthesized by commensal anaerobic bacteria colonized the human gastrointestinal tract such as *P. russellii*, *P. anaerobius*, *P. stomatis*, *C. cadaveris*, *C. sporogenes*, and *C. botulinum*, which belong to the genuses *Peptostreptococcus* and *Clostridium*, and are detectable in the blood plasma of the host (Wlodarska et al., 2017; Roager and Licht, 2018). In our study, serum concentrations of IPA after acute PQ intoxication were significantly lower than those in control group, indicating suppressed commensal bacteria or impaired tryptophan metabolism in gastrointestinal tract caused by PQ intoxication. Thus, we detected six reported IPA-producing bacteria in feces of acute PQ intoxicated mice and control mice. Identification of decreased level of IPA-producing bacteria *C. botulinum* and *P. anaerobius* in feces during acute PQ intoxication could be an explanation for the decreased serum levels of IPA. However, suppression of certain species of gut bacteria by PQ has not been reported yet. Given the high toxicity of PQ itself, we thought that these two bacteria might be more sensitive to PQ toxicity. Besides, we might miss some IPA-producing bacteria which had not been reported. From our results, we inferred that decreased levels of gut bacteria after PQ treatment and successive decreasing of anti-oxidative serum metabolite could be a novel mechanism of PQ toxicity. This result also brought us the probable value of gut microbiota in diagnosis of PQ intoxication where commensal bacteria might become microbe markers.

2-Hydroxybutyric acid was released as a byproduct when cystathionine was cleaved to cysteine which was incorporated into glutathione. Under oxidative stress or detoxification demands, 2-hydroxybutyric acid level increases as the demand of hepatic glutathione synthesis increased (Lord and Bralley, 2008). 2-Hydroxybutyric acid was reported to be an early marker for insulin resistance and impaired glucose regulation, where increased lipid oxidation and oxidative stress were believed to be the probable underlying biochemical mechanisms (Gall et al., 2010). Increasing serum levels of 2-hydroxybutyric acid showed the evidence of oxidative damage in hepatocytes by acute PQ intoxication. Non-essential amino acids serine and glycine biosynthetically linked by serine hydroxymethyltransferases (SHMTs) are essential in biosynthesis of proteins, nucleic acids, and lipids and affect cellular antioxidative capacity through intracellular glutathione biosynthesis (Amelio et al., 2014). *De novo* serine synthesis plays essential roles in cellular anti-oxidative capacity and cell survival when they are exposed to oxidative stress (Riscal et al., 2016; Zhang et al., 2018). Thus, upregulation of 2-hydroxybutyric acid and the ratio of L-serine/glycine in serum of the acute PQ intoxication group compared to the chronic or control group might attribute to oxidative stress caused by PQ redox activity. Combined with ROC analysis of the diagnostic ability, 2-hydroxybutyric acid and the ratio of L-serine/glycine might be valuable in differentiating acute and chronic PQ intoxication.

There were a few limitations in our study that should not be ignored. First, due to the limitation of sensitivity and application of GC-TOF/MS, and the limitation of database ADAP-GC 3.0 we used, we had 43 metabolites remained unknown. Thus, from this study we could only detect a tip of the iceberg of all metabolites. Other analytical platforms, such as nuclear magnetic resonance (NMR) spectroscopy (Bothwell and Griffin, 2011) and LC–MS (Ernst et al., 2014) might be optional complimentary methods in metabolomics study. Second, we were not able to test these biomarkers in human samples to confirm their diagnostic values due to the lack of patient samples. Searching for cooperation with clinical departments to collect patient samples would be our future plan.

## Conclusion

In conclusion, we conducted a serum-based metabolomics profiling analysis in acute and chronic PQ-intoxicated mouse models to monitor metabolic feature. Significant decline of IPA and IPA producing bacteria *C. botulinum* and *P. anaerobius* was identified in acute PQ intoxication group, which could be a mechanism of PQ toxicity and diagnosis candidates; while 2-hydroxybutyric acid and the ratio of L-serine/glycine were shown to be significant metabolite or metabolite ratio for differentiating acute and chronic PQ intoxication. Our results contributed to the better understanding of underlying mechanisms and provided novel diagnostic biomarkers of PQ intoxication.

## Data Availability Statement

The raw data supporting the conclusions of this manuscript will be made available by the authors, without undue reservation, to any qualified researcher.

## Ethics statement

The animal study was reviewed and approved by Institute for Laboratory Animal Research of the Nanjing Medical University.

## Author Contributions

FC was responsible for conceiving, designing the study, supervising the study, and revising the manuscript. YoY, ZG, and ZM wrote the manuscript. YaY, ZM, KL, CC, JL, LH, HF, PC, ZL, CD, HH, YoY, JD, and DL performed the research. ZG analyzed the data. All authors read and approved of the final manuscript.

## Conflict of Interest

The authors declare that the research was conducted in the absence of any commercial or financial relationships that could be construed as a potential conflict of interest.

## Footnotes

1 http://www.hmdb.ca

2 www.kegg.jp

3 https://www.metaboanalyst.ca
