# Supplementary material for: Identification of Serum-Based Metabolic Feature and Characteristic Metabolites in Paraquat Intoxicated Mouse Models
Source: Front Physiol. 2020 Feb 6;11:65. doi: 10.3389/fphys.2020.00065 (PMC7017841; doi:10.3389/fphys.2020.00065)

**Supplemental data**

**Identification of serum-based metabolic feature and characteristic metabolites in paraquat intoxicated mouse models**

Youjia Yu, Zishan Gao, Jiaqian Lou, Zhengsheng Mao, Kai Li, Chunyan Chu, Li Hu, Zheng Li, Chuwei Deng, Hanting Fan, Peng Chen, Huijie Huang, Yanfang Yu, Jingjing Ding, Ding Li, Feng Chen

**Table S1.** Differential metabolites between PQ3d group and ctrl group using univariate statistical analysis.

| Class | Name | HMDB ID | Kegg ID | FC | *P*-value |
| --- | --- | --- | --- | --- | --- |
| Amino Acid | **Ratio of Glycine/L-Serine** | **HMDB00123/HMDB00187** | **C00037/C00065** | **0.565** | **3.50E-04** |
|  | **Ratio of L-Serine/Glycine** | **HMDB00187/HMDB00123** | **C00065/C00037** | **1.774** | **6.00E-04** |
|  | **Ratio of Beta-Alanine/L-Aspartic acid** | **HMDB00056/HMDB00191** | **C00099/C00049** | **0.548** | **2.10E-03** |
|  | **Glycine** | **HMDB00123** | **C00037** | **0.643** | **2.80E-03** |
|  | **Acetylglycine** | **HMDB00532** | **NA** | **8.000** | **4.10E-03** |
|  | **2-Hydroxybutyric acid** | **HMDB00008** | **C05984** | **20.432** | **1.00E-02** |
|  | L-Lysine | HMDB00182 | C00047 | 0.682 | 2.00E-02 |
|  | **Ornithine** | **HMDB00214** | **C00077** | **0.508** | **2.50E-02** |
|  | **Ratio of Sarcosine/Glycine** | **HMDB00271/HMDB00123** | **C00213/C00037** | **3.199** | **2.50E-02** |
|  | **Beta-Alanine** | **HMDB00056** | **C00099** | **0.511** | **3.20E-02** |
|  | Ratio of Urea/L-Arginine | HMDB00294/HMDB00517 | C00086/C00062 | 1.619 | 5.50E-02 |
|  | 5-Hydroxylysine | HMDB00450 | C16741 | 1.520 | 5.90E-02 |
|  | L-Alanine | HMDB00161 | C00041 | 0.789 | 6.00E-02 |
|  | Ratio of L-Tyrosine/L-Phenylalanine | HMDB00158/HMDB00159 | C00082/C00079 | 0.468 | 6.70E-02 |
|  | Methylcysteine | HMDB02108 | NA | 1.531 | 7.90E-02 |
|  | L-Cystine | HMDB00192 | C00491 | 0.324 | 9.00E-02 |
|  | L-Arginine | HMDB00517 | C00062 | 0.561 | 9.80E-02 |
| Carbohydrates | **L-Arabitol** | **HMDB01851** | **C00532** | **0.437** | **4.20E-05** |
|  | **Sorbitol** | **HMDB00247** | **C00794** | **0.057** | **1.30E-03** |
|  | D-Threitol | HMDB04136 | C16884 | 0.751 | 1.60E-02 |
|  | **Rhamnose** | **HMDB00849** | **C00507** | **1.559** | **3.00E-02** |
|  | D-Galactose | HMDB00143 | C00984 | 0.826 | 5.50E-02 |
|  | Mannitol | HMDB00765 | C00392 | 0.661 | 5.90E-02 |
|  | 1,5-Anhydrosorbitol | HMDB02712 | C07326 | 0.715 | 6.90E-02 |
| **Fatty Acids** | **Docosahexaenoic acid** | **HMDB02183** | **C06429** | **2.083** | **9.40E-03** |
|  | **Oleic acid** | **HMDB00207** | **C00712** | **3.204** | **9.80E-03** |
|  | **Linoleic acid** | **HMDB00673** | **C01595** | **2.159** | **1.30E-02** |
|  | **Myristic acid** | **HMDB00806** | **C06424** | **2.663** | **4.00E-02** |
| **Indoles** | **3-Indolepropionic acid** | **HMDB02302** | **NA** | **0.228** | **1.20E-04** |
| **Lipids** | **Cholesterol** | **HMDB00067** | **C00187** | **1.789** | **1.00E-02** |
|  | O-Phosphoethanolamine | HMDB00224 | C00346 | 1.135 | 6.70E-02 |
| **Nucleotide** | **Guanosine** | **HMDB00133** | **C00387** | **0.263** | **2.20E-03** |
|  | **Uracil** | **HMDB00300** | **C00106** | **2.812** | **1.80E-02** |
|  | Ratio of Inosine/Adenosine | HMDB00195/HMDB00050 | C00294/C00212 | 4.491 | 5.90E-02 |
|  | Inosine | HMDB00195 | C00294 | 6.474 | 6.00E-02 |
| **Organic Acids** | **Hypotaurine** | **HMDB00965** | **C00519** | **0.548** | **2.10E-02** |
|  | **2-Hydroxy-3-methylbutyric acid** | **HMDB00407** | **NA** | **5.028** | **2.30E-02** |
|  | **L-Lactic acid** | **HMDB00190** | **C00186** | **1.841** | **4.00E-02** |
|  | Ratio of Pyruvic acid/L-Lactic acid | HMDB00243/HMDB00190 | C00022/C00186 | 0.362 | 6.30E-02 |
|  | Ratio of Oxoglutaric acid/Isocitric acid | HMDB00208/HMDB00193 | C00026/C00311 | 0.490 | 7.00E-02 |
|  | Oxalic acid | HMDB02329 | C00209 | 1.355 | 7.20E-02 |
|  | Glycolic acid | HMDB00115 | C00160 | 0.800 | 7.60E-02 |

Bolded metabolites with significant *P*-value < 0.05 and > 1.5 fold increase or decrease.

*P*-values were calculated from the Student’s *t*-test.

**Table S2.** Differential metabolites between PQ30d group and ctrl group using univariate statistical analysis.

| Class | Name | HMDB ID | Kegg ID | FC | *P*-value |
| --- | --- | --- | --- | --- | --- |
| Alcohols | 2-Hydroxypyridine | HMDB13751 | C02502 | 1.088 | 2.00E-02 |
| Amino Acid | **Ratio of L-Tyrosine/L-Phenylalanine** | **HMDB00158/HMDB00159** | **C00082/C00079** | **1.531** | **4.10E-04** |
|  | **Ratio of L-Valine/Alpha-ketoisovaleric acid** | **HMDB00883/HMDB00019** | **C00183/C00141** | **0.576** | **7.80E-03** |
|  | **L-Leucine** | **HMDB00687** | **C00123** | **0.649** | **8.80E-03** |
|  | L-Valine | HMDB00883 | C00183 | 0.673 | 1.00E-02 |
|  | L-Alloisoleucine | HMDB00557 | NA | 0.691 | 1.50E-02 |
|  | **Ratio of Alpha-ketoisovaleric acid/L-Valine** | **HMDB00019/HMDB00883** | **C00141/C00183** | **1.738** | **1.60E-02** |
|  | **Aminomalonic acid** | **HMDB01147** | **C00872** | **1.722** | **1.90E-02** |
|  | Alpha-ketoisovaleric acid | HMDB00019 | C00141 | 1.425 | 5.50E-02 |
|  | L-Lysine | HMDB00182 | C00047 | 0.795 | 7.10E-02 |
|  | L-Proline | HMDB00162 | C00148 | 0.683 | 8.50E-02 |
| Carbohydrates | Threonic acid | HMDB00943 | C01620 | 1.202 | 3.10E-02 |
|  | Mannitol | HMDB00765 | C00392 | 1.534 | 6.10E-02 |
| Lipids | **MG182** | **HMDB11568** | **NA** | **1.970** | **1.60E-02** |
| Nucleotide | Inosine | HMDB00195 | C00294 | 6.992 | 9.70E-02 |
| Organic Acids | Pyruvic acid | HMDB00243 | C00022 | 0.731 | 1.90E-02 |
|  | Isocitric acid | HMDB00193 | C00311 | 1.369 | 3.60E-02 |
|  | Glyceric acid | HMDB00139 | C00258 | 1.063 | 6.70E-02 |
|  | Citric acid | HMDB00094 | C00158 | 1.814 | 7.40E-02 |

Bolded metabolites with significant *P*-value < 0.05 and >1.5 fold increase or decrease.

*P*-values were calculated from the Student’s *t*-test.

**Table S3.** Differential metabolites between PQ3d group and PQ30d group using univariate statistical analysis.

| Class | Name | HMDB ID | Kegg ID | FC | *P*-value |
| --- | --- | --- | --- | --- | --- |
| Alcohols | 2-Hydroxypyridine | HMDB13751 | C02502 | 0.801 | 9.20E-02 |
| Amino Acid | **Ratio of L-Serine/Glycine** | **HMDB00187/HMDB00123** | **C00065/C00037** | **2.031** | **8.70E-05** |
|  | **Ratio of Glycine/L-Serine** | **HMDB00123/HMDB00187** | **C00037/C00065** | **0.494** | **4.80E-04** |
|  | **Ratio of L-Tyrosine/L-Phenylalanine** | **HMDB00158/HMDB00159** | **C00082/C00079** | **0.306** | **1.50E-03** |
|  | **Ratio of L-Valine/Alpha-ketoisovaleric acid** | **HMDB00883/HMDB00019** | **C00183/C00141** | **1.578** | **5.70E-03** |
|  | **L-Leucine** | **HMDB00687** | **C00123** | **1.783** | **2.70E-02** |
|  | Glycine | HMDB00123 | C00037 | 0.676 | 4.00E-03 |
|  | **Acetylglycine** | **HMDB00532** | **NA** | **8.338** | **4.70E-03** |
|  | **2-Hydroxybutyric acid** | **HMDB00008** | **C05984** | **24.915** | **9.90E-03** |
|  | Ratio of Oxoglutaric acid/L-Glutamic acid | HMDB00208/HMDB00148 | C00026/C00025 | 0.681 | 2.70E-02 |
|  | **Beta-Alanine** | **HMDB00056** | **C00099** | **0.552** | **3.00E-02** |
|  | Ratio of Beta-Alanine/L-Aspartic acid | HMDB00056/HMDB00191 | C00099/C00049 | 0.699 | 3.10E-02 |
|  | **L-Valine** | **HMDB00883** | **C00183** | **1.527** | **4.60E-02** |
|  | L-Alloisoleucine | HMDB00557 | NA | 1.323 | 4.30E-02 |
|  | **Ratio of Alpha-ketoisovaleric acid/L-Valine** | **HMDB00019/HMDB00883** | **C00141/C00183** | **0.638** | **3.50E-02** |
|  | **L-Cystine** | **HMDB00192** | **C00491** | **0.351** | **3.60E-02** |
|  | **Ratio of Urea/L-Arginine** | **HMDB00294/HMDB00517** | **C00086/C00062** | **2.001** | **3.70E-02** |
|  | **L-Phenylalanine** | **HMDB00159** | **C00079** | **1.537** | **4.30E-02** |
|  | L-Cysteine | HMDB00574 | NA | 0.706 | 4.60E-02 |
|  | Ratio of Sarcosine/Glycine | HMDB00271/HMDB00123 | C00213/C00037 | 2.481 | 5.50E-02 |
|  | Ratio of L-Glutamic acid/Oxoglutaric acid | HMDB00148/HMDB00208 | C00025/C00026 | 1.463 | 5.90E-02 |
|  | Urea | HMDB00294 | C00086 | 1.513 | 7.60E-02 |
| Carbohydrates | **Sorbitol** | **HMDB00247** | **C00794** | **0.055** | **1.00E-03** |
|  | **L-Arabitol** | **HMDB01851** | **C00532** | **0.489** | **2.70E-03** |
|  | **Mannitol** | **HMDB00765** | **C00392** | **0.431** | **1.20E-02** |
| Fatty Acids | **Linoleic acid** | **HMDB00673** | **C01595** | **2.152** | **1.60E-02** |
|  | **Oleic acid** | **HMDB00207** | **C00712** | **2.892** | **2.50E-02** |
|  | **Docosahexaenoic acid** | **HMDB02183** | **C06429** | **1.620** | **4.20E-02** |
|  | Arachidonic acid | HMDB01043 | C00219 | 1.536 | 7.30E-02 |
|  | Myristic acid | HMDB00806 | C06424 | 2.205 | 9.00E-02 |
| Indols | **3-Indolepropionic acid** | **HMDB02302** | **NA** | **0.205** | **1.30E-03** |
|  | Serotonin | HMDB00259 | C00780 | 0.638 | 8.00E-02 |
| Lipids | **MG182** | **HMDB11568** | **NA** | **0.324** | **3.20E-03** |
|  | **Cholesterol** | **HMDB00067** | **C00187** | **1.612** | **1.70E-02** |
|  | Glycerol 3-phosphate | HMDB00126 | C00093 | 0.722 | 1.80E-02 |
| Nucleotide | **Uracil** | **HMDB00300** | **C00106** | **2.734** | **1.00E-02** |
|  | **Guanosine** | **HMDB00133** | **C00387** | **0.284** | **2.20E-02** |
|  | Adenosine | HMDB00050 | C00212 | 1.547 | 5.00E-02 |
| Organic Acids | **Ratio of Fumaric acid/Succinic acid** | **HMDB00134/HMDB00254** | **C00122/C00042** | **0.483** | **8.40E-04** |
|  | **2-Hydroxy-3-methylbutyric acid** | **HMDB00407** | **NA** | **3.373** | **3.20E-02** |
|  | Succinic acid | HMDB00254 | C00042 | 2.184 | 6.10E-02 |
|  | Malic acid | HMDB00744 | C00711 | 0.638 | 7.80E-02 |
|  | Hypotaurine | HMDB00965 | C00519 | 0.669 | 8.40E-02 |
|  | L-Lactic acid | HMDB00190 | C00186 | 1.603 | 8.60E-02 |
| Vitamin | **Pantothenic acid** | **HMDB00210** | **C00864** | **0.330** | **4.70E-03** |

Bolded metabolites with significant *P*-value < 0.05 and >1.5 fold increase or decrease.

*P*-values were calculated from the Student’s *t*-test.

**Table S4.** Comparison results of PQ 3d group vs ctrl group by Bonferroni correction or FDR correction following *t*-test and Lasso regression.

| **Bonferroni correction** | ***P*-value** |
| --- | --- |
| 3-Indolepropionic.acid | 0.04001822 |
| **FDR correction** | **Adjusted *P*-value** |
| 2.Hydroxybutyric.acid | 0.0004159187 |
| Glycine | 0.0023082716 |
| Acetylglycine | 0.0031099548 |
| L.Arabitol | 0.0004372888 |
| Oleic.acid | 0.0026464254 |
| 3.Indolepropionic.acid | 0.0002286756 |
| Inosine | 0.0013022046 |
| 2.Hydroxy.3.methylbutyric.acid | 0.0034191633 |
| Ratio.of.Beta.Alanine./L.Aspartic.acid | 0.0020643432 |
| Ratio.of.L.Serine./Glycine | 0.0006006160 |
| Ratio.of.Glycine./L.Ser | 0.0003512792 |
| **Lasso regression** | **Coefficients** |
| 2.Hydroxybutyric.acid | 0.193785404 |
| L.Lysine | -0.691470557 |
| L.Arabitol | -0.004685646 |
| 3.Indolepropionic.acid | -0.766348084 |
| 2.Hydroxy.3.methylbutyric.acid | 0.000835890 |
| Ratio.of.Glycine. /L.Serine | -0.468387540 |

**Table S5.** Comparison results of PQ 30d group vs ctrl group by Lasso regression.

| **Lasso regression** | **Coefficients** |
| --- | --- |
| L.Valine | -0.036519100 |
| Aminomalonic.acid | 0.327417951 |
| Threonic.acid | 0.095408105 |
| Inosine | 0.423017363 |
| Isocitric.acid | 0.198673323 |
| Ratio.of.L.Tyrosine./L.Phenylalanine | 0.794599030 |

**Table S6.** Comparison results of PQ 3d group vs PQ 30d group by Bonferroni correction or FDR correction following *t*-test and Lasso regression.

| **Bonferroni correction** | ***P*-value** |
| --- | --- |
| 3-Indolepropionic.acid | 0.02366588 |
| Ratio.of.L.Serine./Glycine | 0.01520688 |
| **FDR correction** | **Adjusted *P*-value** |
| 2.Hydroxybutyric.acid | 3.488447e-04 |
| Glycine | 1.251415e-03 |
| L.Arabitol | 1.462788e-03 |
| Mannitol | 4.317037e-04 |
| Sorbitol | 3.527095e-03 |
| 3.Indolepropionic.acid | 1.352336e-04 |
| Pantothenic.acid | 2.651249e-03 |
| Ratio.of.L.Serine./Glycine | 8.689643e-05 |
| Ratio.of.Glycine./L.Serine | 4.790217e-04 |
| Ratio.of.L.Tyrosine./L.Phenylalanine | 1.495456e-03 |
| Ratio.of.Fumaric.acid./Succinic.acid | 8.399055e-04 |
| **Lasso regression** | **Coefficients** |
| 2.Hydroxybutyric.acid | 1.70125514 |
| Glycine | -0.26502190 |
| Pantothenic.acid | -0.07054445 |
| Ratio.of.L.Serine./Glycine | 1.43264376 |
| Ratio.of.Fumaric.acid./Succinic.acid | -0.02031136 |

**Table S7.** Results from pathway analysis of PQ 3d group vs ctrl group.*

| pathway | Total | Expected | Hits | Raw *P* | -log(*P*) | Holm adjust | FDR | Impact |
| --- | --- | --- | --- | --- | --- | --- | --- | --- |
| **Glycine, serine and threonine metabolism** | **48** | **0.15953** | **3** | **0.000389** | **7.8524** | **0.030716** | **0.015552** | **0.32378** |
| Aminoacyl-tRNA biosynthesis | 75 | 0.24927 | 3 | 0.001455 | 6.5331 | 0.11345 | 0.038786 | 0.11268 |
| Pantothenate and CoA biosynthesis | 27 | 0.089738 | 2 | 0.003256 | 5.7274 | 0.25068 | 0.056003 | 0.07286 |
| beta-Alanine metabolism | 28 | 0.093062 | 2 | 0.0035 | 5.6549 | 0.26601 | 0.056003 | 0.25694 |
| Methane metabolism | 34 | 0.113 | 2 | 0.005143 | 5.2701 | 0.38572 | 0.062234 | 0.01751 |
| Propanoate metabolism | 35 | 0.11633 | 2 | 0.005446 | 5.213 | 0.40297 | 0.062234 | 0.085 |
| Nitrogen metabolism | 39 | 0.12962 | 2 | 0.006737 | 5.0002 | 0.49177 | 0.067366 | 0.00067 |
| Cysteine and methionine metabolism | 56 | 0.18612 | 2 | 0.013608 | 4.2971 | 0.97978 | 0.12096 | 0.01197 |
| Purine metabolism | 92 | 0.30577 | 2 | 0.034823 | 3.3575 | 1 | 0.27859 | 0.00425 |

**Table S8.** Results from pathway analysis of PQ 30d group vs ctrl group.*

| pathway | Total | Expected | Hits | Raw *P* | -log(*P*) | Holm adjust | FDR | Impact |
| --- | --- | --- | --- | --- | --- | --- | --- | --- |
| Phenylalanine, tyrosine and tryptophan biosynthesis | 27 | 0.078521 | 2 | 0.002459 | 6.0081 | 0.19424 | 0.098347 | 0.008 |
| Phenylalanine metabolism | 45 | 0.13087 | 2 | 0.006763 | 4.9963 | 0.52075 | 0.13526 | 0.11906 |

*Metabolic pathways with Raw *P* > 0.05, Impact factor > 0 were listed.

**Figure S1.** Correlation analysis of PQ3d and PQ30d showed positive correlation between 2-hydroxybutyric acid and Ratio of L.serine/glycine.


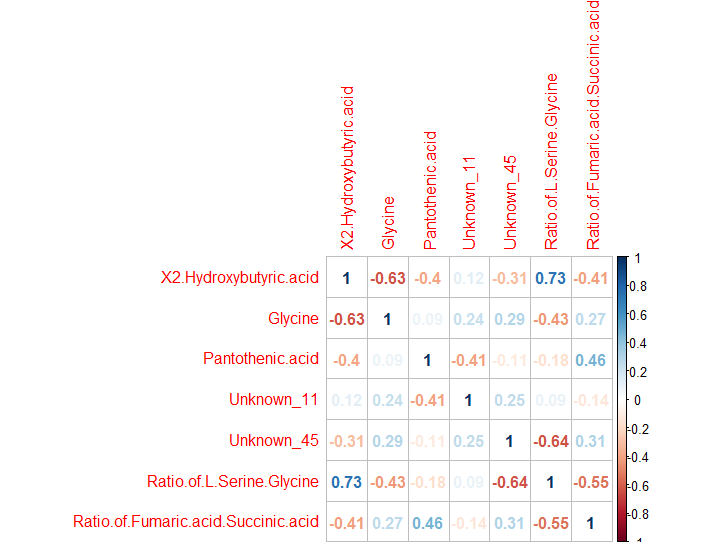

Supplement: Supplementary file 2 [file Data_Sheet_1.docx]
